# The human immune response to tuberculosis and its treatment: a view from the blood

**DOI:** 10.1111/imr.12269

**Published:** 2015-02-20

**Authors:** Jacqueline M Cliff, Stefan H E Kaufmann, Helen McShane, Paul van Helden, Anne O'Garra

**Affiliations:** 1 TB Centre and Department of Immunology and Infection, London School of Hygiene & Tropical MedicineLondon, UK; 2 Department of Immunology, Max Planck Institute for Infection BiologyBerlin, Germany; 3 The Jenner Institute, University of OxfordOxford, UK; 4 DST/NRF Centre of Excellence for Biomedical Tuberculosis Research/MRC Centre for Tuberculosis Research, Division of Molecular Biology and Human Genetics, Faculty of Medicine and Health Sciences, Stellenbosch UniversityStellenbosch, South Africa; 5 Division of Immunoregulation, MRC National Institute for Medical ResearchLondon, UK; 6 NHLI, Faculty of Medicine, Imperial College LondonLondon, UK

**Keywords:** tuberculosis, transcriptional signature, immune response

## Abstract

The immune response upon infection with the pathogen *Mycobacterium tuberculosis* is poorly understood, hampering the discovery of new treatments and the improvements in diagnosis. In the last years, a blood transcriptional signature in tuberculosis has provided knowledge on the immune response occurring during active tuberculosis disease. This signature was absent in the majority of asymptomatic individuals who are latently infected with *M. tuberculosis* (referred to as latent). Using modular and pathway analyses of the complex data has shown, now in multiple studies, that the signature of active tuberculosis is dominated by overexpression of interferon-inducible genes (consisting of both type I and type II interferon signaling), myeloid genes, and inflammatory genes. There is also downregulation of genes encoding B and T-cell function. The blood signature of tuberculosis correlates with the extent of radiographic disease and is diminished upon effective treatment suggesting the possibility of new improved strategies to support diagnostic assays and methods for drug treatment monitoring. The signature suggested a previously under-appreciated role for type I interferons in development of active tuberculosis disease, and numerous mechanisms have now been uncovered to explain how type I interferon impedes the protective response to *M. tuberculosis* infection.

## Introduction

The immune response underlying protection or pathogenesis in tuberculosis is incompletely understood, hindering the development of new vaccines and therapeutics (reviewed in 1). A third of the world is estimated to be infected with *Mycobacterium tuberculosis*; however, only 10–20% of individuals go on to develop active disease during their lifetime, the rest remaining asymptomatic are referred to as ‘latent’ (reviewed in 2). Furthering our understanding of the immune response would be helpful in determining why some people exposed to *M. tuberculosis* remain latent and others go to develop disease. Such knowledge will also be useful for the development of vaccines and therapeutics and may provide information required to support the identification of diagnostic and prognostic markers. Although *M. tuberculosis* antigen-specific interferon-gamma (IFN-γ) release assays can determine whether individuals have been infected with *M. tuberculosis*, they cannot distinguish individuals with active tuberculosis and those latently infected with *M. tuberculosis*. This determination relies on assessment of clinical symptoms and mycobacterium sputum smear positivity, neither of which are highly specific. Confirmatory diagnosis of tuberculosis requires culture of the mycobacteria for speciation, which alternatively can now be achieved using the nucleic acid amplification testing (NAAT). Both are reliant on obtaining sputum from infected individuals, which is not always feasible, or bronchoalveolar lavage, which requires an invasive technique not easily accessible in countries with a high tuberculosis burden. Therefore, the ability to determine the immune response in active tuberculosis using blood transcriptomics (3–10) offers new opportunities to develop supportive biomarkers to determine whether individuals suffer from active tuberculosis. The diminished blood transcriptomic signature observed during successful tuberculosis treatment (4, 11, 12) could also help in monitoring the response to treatment and in the development of new drugs, since current tests for monitoring drug efficacy such as the early bactericidal assays and 2-month sputum conversion remain inadequate (13, 14). The immune response uncovered using blood transcriptomics of active tuberculosis has revealed hitherto unappreciated knowledge, which may also be of great use in the development of therapeutics.

## A view of the immune response from the blood using transcriptomics

Peripheral blood is easily accessible and represents a reservoir of immune cells trafficking to and from the sites of active disease and lymphoid organs. The use of peripheral blood for determining gene expression profiles in disease was first introduced in studies of cancer (15–18) and then autoimmune pathologies (19). More recently the blood transcriptome has been of success in measuring the immune response to infection (reviewed in 5) and can reflect disease activity in the tissue (4). It has been shown that in some cases blood transcriptional signatures may help to distinguish different infectious diseases, such as bacteria or viruses, but can also distinguish between different bacterial or viral infections (5, 20). In some cases, however, similar transcriptional signatures can be observed in certain types of diseases with similar pathogenesis (21–23) or infectious agents (24). The specificity of the transcriptional signature for a particular infectious disease requires a combination of activated pathways and transcription programs, rather than a set of uniquely activated disease-specific genes. Pathway and modular bioinformatics analyses have been designed to characterize a transcriptional response to identify combinations of pathways and networks associated with diseases (19, 25). Such approaches have been applied successfully to the study of the immune response in tuberculosis (4, 12, 21). Methods for quantitation of transcriptomic profiles to reflect disease severity, such as the molecular distance to health (MDTH) (25) or variations on this approach, have been successfully developed and can also be used to quantify the disease response to treatment (4, 11, 26).

## Overview of transcriptomic studies in human tuberculosis

### Discrimination of active tuberculosis from latent infection

Strategies to improve and/or support current diagnostic tests for active tuberculosis would be of great use in clinical management of the disease. The ability to provide an earlier diagnosis of the disease would allow more rapid treatment initiation, which could reduce the risk of transmission. Support and improvement of current diagnostic tests would also minimize misdiagnosis of other confounding diseases as tuberculosis and would thus avoid the adverse events resulting from unnecessary anti-tuberculosis treatment. This will be particularly useful for diagnosis of tuberculosis in hard to diagnose groups like infants and human immunodeficiency virus (HIV)-infected individuals.

**Figure 1:**
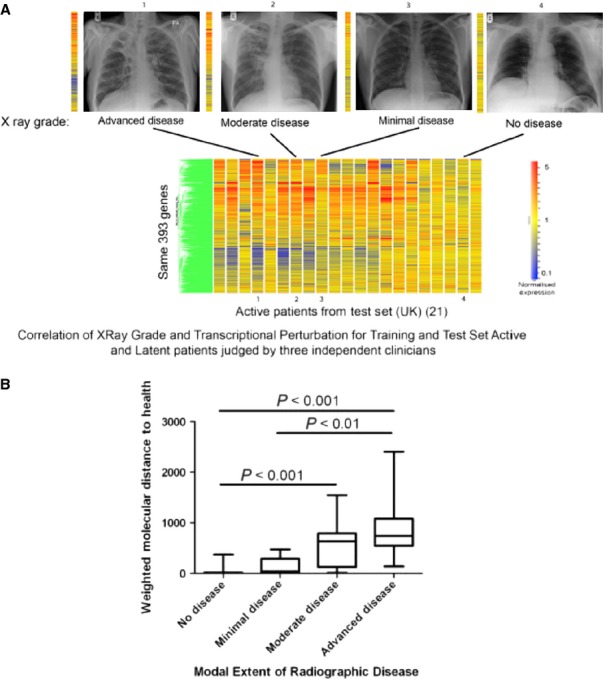
The blood transcriptional signature of active tuberculosis correlates with radiographic extent of disease in the lung. Hierarchical clustering analysis and statistical filtering generated a gene tree (green, left of figure) of 393 transcripts, and an expression profile (vertical columns) (4) (A) here shown for selected participants correlating with lung radiographic extent of disease. Each row of the heatmap represents an individual transcript and each column an individual participant. Relative abundance of transcripts is shown as: over-abundant red; under-abundant blue; median colored yellow. (B) The Weighted Molecular Distance to Health calculated for the transcriptional signature is shown against the score for the radiographic extent of disease. Adapted from Berry *et al*., 2010, *Nature* (4).

Several studies have reported the blood transcriptome of patients with active tuberculosis (3, 5, 6) (*Table* 1). Early studies in patients with active tuberculosis indicated that differences existed between their blood gene expression profiles and those in healthy, latently infected individuals (27, 28). These early studies identified immune response genes and chemokine genes to be differentially expressed in active pulmonary tuberculosis patients and controls, but involved small numbers of patients and required further validation. In 2010, a larger scale study was conducted, including patients recruited in both London, U.K. and Cape Town, South Africa, all recruited before initiation of treatment (4). Whole genome blood transcriptomic profiles clearly discriminated individuals with active tuberculosis from those latently infected with *M. tuberculosis* and healthy controls (4). Furthermore, the magnitude of the transcriptional signature correlated with the extent of disease in the lungs as measured by radiography (4) (*Fig*.1), indicating that the immune response in the blood can reflect the local reaction to a pathogen in the lung. The observed transcriptional signature was replicated in independent and validation cohorts (4). The blood transcriptional signature, as defined by various approaches including pathway and modular analysis, was shown to consist of upregulated IFN-inducible genes (both type I and type II), myeloid and inflammatory genes, and downregulated transcripts encoding B and T-cell genes (4). Flow cytometric analysis together with purification of leukocyte populations from the blood of identical individuals revealed that the downregulation of T-cell genes was largely due to diminished numbers of subsets of CD4^+^ T-cell effector and memory cells (4), possibly reflecting apoptosis and/or migration to the infected lung. A parallel study also demonstrated a blood transcriptional signature that could distinguish patients with active tuberculosis from latently infected individuals (8). Here, Fcγ receptor 1B (FCGRIB) was defined as the most differentially expressed gene and in combination with four other markers gave high specificity for discrimination of active tuberculosis from individuals with latent infection and healthy controls. Elevated expression of innate immune-related genes, including Janus kinase (JAK)-STAT pathway, sensing of microbial patterns by Toll-like receptors, and IFN signaling was also observed in active tuberculosis and suggested a high degree of accuracy in discriminating tuberculosis patients and latently infected donors and that expression of these genes correlated with susceptibility and resistance to tuberculosis (8).

**Table 1:** Different studies demonstrating use of transcriptional blood signatures to identify patients with active tuberculosis

Geographical region	Year	Sample	Study design	Key pathways	Reference
South Africa	2007	Whole blood	TB versus LTB	–	(28)
Germany	2007	PBMC	TB versus LTB	Fcγ-receptor signalling	(27)
UK and South Africa	2010	Whole blood	TB versus LTB versus HC, TB versus OD, TB treatment	IFN signaling	(4)
The Gambia	2011	Whole blood	TB versus LTB	JAK–STAT pathway; IFN signaling; TLR	(7)
USA & Brazil	2011	Whole blood	TB versus LTB versus HC	Interferon signaling	(29)
South Africa	2011	Whole blood	TB versus LTB versus HC	–	(8)
Germany	2012	Whole blood	TB versus OD	IFN signaling; Complement; TLR signaling; Fcγ-receptor signalling	(44)
South Africa	2012	Whole blood	TB treatment	–	(11)
Indonesia	2012	PBMC	TB versus HC, TB treatment	IFN signaling	(10)
South Africa, Malawi	2013	Whole blood	TB versus OD, TB versus LTB	–	(30)
UK	2013	Whole Blood	TB versus OD, TB treatment	IFN signaling, Role of pattern recognition receptors, Antigen presentation	(21)
South Africa	2013	Whole blood	TB treatment	Complement; B-cell markers; CD64	(12)
South Africa, Kenya, Malawi	2014	TB versus OD, TB versus LTB	–		(36)

**Figure 2:**
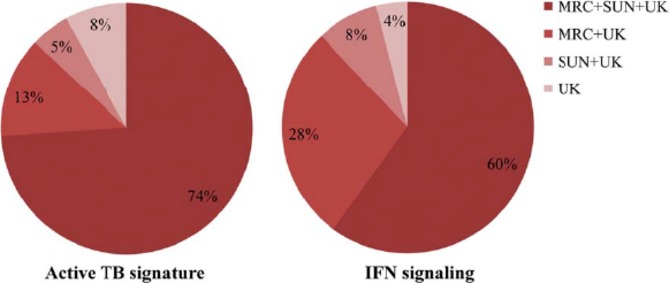
Comparison of differential expression in three independent cohorts. Comparison of differential gene expression between active tuberculosis (TB) and individuals with latently infected with *M. tuberculosis* (LTBI) in cohorts in South Africa (SUN) and The Gambia (MRC) with gene profiles in a cohort in the United Kingdom (UK) and South Africa identified by Berry *et al*. (4). Significance of differential expression at corrected p-value level of q  < 0.05. From Maertzdorf *et al*., 2011, *Plos One* (7).

Similarity between these two independent studies (4, 8) was subsequently reported (7). Such whole blood gene expression in tuberculosis patients, as well as in healthy infected and uninfected individuals was also analyzed in an additional cohort in The Gambia, West Africa and validated against the previously identified signatures (4, 8, 27) (*Fig*.2). A unique combination of classical gene expression analysis with pathway and functional association analysis integrated with intra-individual expression correlations was used and revealed high similarities of expression profiles among different cohorts (7) (*Fig*.2). This study identified a network of Fcγ receptor 1 signaling correlating with transcriptional activity as the hallmark of gene expression in tuberculosis (7) and validated the previously reported (4, 8) functional gene clusters of immunoregulatory interactions involving the JAK-STAT pathway, sensing of microbial patterns by Toll-like receptors and IFN signaling. Collectively, these reports first suggested that the use of blood transcriptomics could provide a robust system for identification and validation of biosignatures for tuberculosis, which could support its diagnosis. In addition, the use of integrated and novel bioinformatic analysis tools (4, 8) to interrogate the blood transcriptional signature revealed novel insights into functional immune and inflammatory networks underlying the pathogenesis of tuberculosis.

Further studies describing discriminatory gene expression profiles, which segregate individuals with active tuberculosis from those asymptomatic individuals latently infected with *M. tuberculosis* or from uninfected healthy control subjects, have subsequently been published by several different research groups. These studies have been conducted using a variety of microarray platforms, and importantly patients have been recruited in different geographical locations, including Brazil (29), The Gambia (7), Indonesia (10), South Africa, and Malawi (30), again supporting the robustness of using whole blood gene expression profiles in the identification of individuals with active tuberculosis as distinct from those latently infected individuals and healthy controls. Although initially there were concerns that discrimination might be masked unless cell subsets were characterized separately, the IFN-inducible signature identified by Berry *et al*. (4) was clearly observable directly in whole blood from active tuberculosis patients versus controls.

The total transcript composition of whole blood can indicate important differences in cell populations. Berry *et al*. (4) reported that the whole blood IFN-inducible signature of active tuberculosis was observed in purified neutrophils from the same individuals, with a subset of the genes also upregulated in purified monocytes. The blood transcriptome of active tuberculosis showed a decrease in B-cell and T-cell-specific genes (4), and flow cytometric analysis of the blood from these patients revealed that the T-cell signature could be explained by decreases in central and effector memory T cells in the blood of active tuberculosis patients (4). This is in keeping with accumulating evidence that the monocyte/lymphocyte ratio is associated with the risk of developing active tuberculosis following infection (31) and has been observed in whole blood transcriptomic studies (Fletcher, personal communication). Nevertheless, gene expression profiling of selected CD4^+^ and CD8^+^ T cells provided a highly discriminatory gene expression profile for active tuberculosis versus latent infection (32). The identified cytokine receptor signaling regulation included Interleukin (IL-2) receptor α-chain, JAK3, suppressor of cytokine signaling 3 (SOCS3), cytokine-inducible SH2-containing protein, and protoncogene serine/threoinine protein kinase. It needs to be clarified whether this regulatory signaling network indeed contributes to impaired T-cell responses in tuberculosis.

A comparison (6) of eight independently obtained tuberculosis microarray datasets (4, 7, 8, 10, 12, 23, 27, 28) revealed enrichment for genes associated with myeloid cell inflammation and TREM1 signaling as the most significant pathways upon integration of data from all the studies (6) rather than the IFN signaling pathway (4, 8, 10). This signature of myeloid inflammation had also been observed in earlier studies using the modular approach (4, 12). Flow cytometric analysis of leukocyte subsets isolated from the blood of the same active tuberculosis patients indicated that the pronounced myeloid signature resulted from increased numbers of inflammatory monocytes and increased gene expression in purified monocytes (4). True dominance of a particular pathway will require quantitative analysis by other more sophisticated approaches in the future. It is possible, however, that in future in depth meta-analysis of the active tuberculosis blood signature, when quantitatively compared and including a larger set of confounding diseases in increased numbers of individuals, may reveal yet another dominant pathway or set of genes better able to distinguish tuberculosis from other diseases and of use in diagnosis. Regardless, it is likely that the different pathways of genes perturbed during active tuberculosis may all contribute to pathogenesis of the disease, such as the IFN-inducible genes described in the blood signature of active tuberculosis (4, 7, 8, 10, 12).

### Discrimination of active tuberculosis from other confounding diseases

Remarkable similarities have been reported in the blood transcriptome of individuals with tuberculosis and the granulomatous disease sarcoidosis (21–23). Sarcoidosis is a multi-system granulomatous disease, predominantly a respiratory disorder of unknown etiology, which presents with very similar radiological and clinical findings to tuberculosis. Both diseases were found to have significant overlap in the differentially expressed genes in the blood, including the IFN-inducible genes (21–23), indicating that common pathways may result in the similar pathological features observed in both diseases. In keeping with this theory, another bacterial granulomatous disease affecting the lung, melioidosis, also revealed some overlap with the IFN-dominated blood transcriptional signature of pulmonary tuberculosis (33). On the other hand, patients with community-acquired pneumonia and lung cancer were shown to display a distinct blood transcriptional signature to that seen in tuberculosis, which instead is dominated by an inflammatory signature (21) and not the dominant IFN-inducible signature of tuberculosis and sarcoidosis. Transcript classifiers that discriminated between tuberculosis were identified (21–23).

Around a quarter of all tuberculosis deaths occur in HIV-infected people who often test negative for mycobacteria, e.g. sputum smear negative (34), hence the ability to diagnose active tuberculosis in this group is vital for improved patient care and tuberculosis control programs. The recent identification of gene expression signatures that can distinguish individuals with active tuberculosis from those with other lung diseases and in *M. tuberculosis* infection in HIV^+^ individuals is very encouraging (30). A large case–controlled study, involving both HIV-infected and HIV-uninfected individuals, prospectively recruited patients presenting with symptoms of tuberculosis, some of whom on further investigation were diagnosed with another disease and not tuberculosis. This study generated a large clinically relevant and diverse cohort of confounding diseases to act as a comparison group with the positively identified tuberculosis cohorts (30). The derived transcriptional signatures were able to differentiate, with a high degree of sensitivity and specificity, between tuberculosis and healthy controls as well as between tuberculosis and other confounding diseases in both HIV-infected and HIV-uninfected individuals (30). Pediatric tuberculosis is notoriously difficult to diagnose and often depends exclusively on clinical symptoms (35). The characterization of an altered blood gene expression profile in children with active tuberculosis comprising both HIV-infected and HIV-uninfected individuals (36) provides new leads for the development of new, host-based tests, to support the diagnosis of tuberculosis in pediatric disease (36) in addition to adult disease (4, 7, 8, 10, 12, 21, 30).

### What is needed for blood transcriptomics to support diagnostics or prognostics?

To develop this approach and to verify proposed classifiers as a support to current diagnostics, testing in further independent cohorts will be necessary to evaluate diagnostic accuracy, and additionally it will be necessary to validate transcript classifiers in a range of independent cohorts of respiratory and systemic illnesses. A dominant algorithm of transcript classifiers, consisting of several sets of ca. 12–100 genes associated with active tuberculosis, may be the best approach for the development of a diagnostic with the highest specificity and sensitivity, and this approach would be required considering current costs in order to be of use in the clinic. Yet, as technology advances for processing and gene expression analysis together with advances in analytical tools (37) and also becomes more affordable, it is possible that a diagnosis may be based on the whole set of genes discriminating tuberculosis from other diseases and on selected classifier genes. The use of blood transcriptomics to improve diagnosis of tuberculosis could be applied together with current methods including clinical symptoms, sputum mycobacterium smear positivity, and confirmatory diagnosis, where possible, of tuberculosis by culture or NAAT technology. This will be especially valuable in cases who are smear negative or culture negative, and in children where such tests are not always feasible. Blood transcriptomics may help to speed up diagnosis and predict which people have disease, allowing faster treatment and thus reduction of transmission.

Berry *et al*. (4) reported that the signature of active tuberculosis was detectable in 10–20% of asymptomatic individuals diagnosed with latent *M. tuberculosis* infection [by the IGRA test, an *M. tuberculosis* specific antigen-stimulated IFN-γ release assay of human blood or the tuberculin skin test (TST)-skin test], although this could be reflective of subclinical tuberculosis at early stages of disease. This needs to be verified in larger number of latently infected individuals, and it will be of interest to determine whether those with a signature of tuberculosis go on to develop disease or whether their signature reverts to that of healthy controls and the majority of latently infected individuals. This would be in keeping with the heterogeneity in individuals who are latently infected with *M. tuberculosis* that has been reported using high-resolution computed tomography (CT) and positron emission tomography (PeT) (PET-CT) (2). Currently, data from a longitudinal study which followed household contacts of newly diagnosed tuberculosis patients over several years is being analyzed. In this study, blood transcriptome profiles are compared in those household contacts who remained healthy over the observational period versus those who developed active TB. It is hoped that this study will provide a first hint about a biosignature that can predict individuals at heightened risk of developing active tuberculosis disease (9, 13, 38) (http://www.biomarkers-for-tb.net/). Another ongoing study determines risk of tuberculosis disease in adolescents living in a TB-high-endemic area (39, 40).

## Changes in blood gene expression during tuberculosis treatment

**Figure 3:**
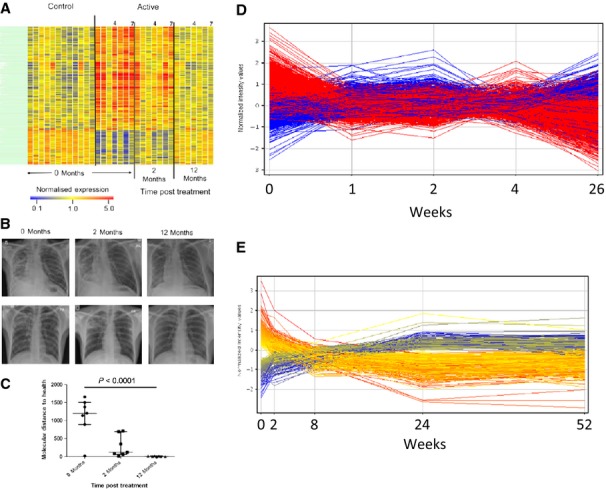
The blood transcriptional signature of tuberculosis is diminished upon successful treatment. (A) The transcriptional signature of active TB is extinguished during treatment (4). (B) Transcriptional profiles of Active TB patients, resampled at 2 and 12 months postantimycobacterial drug treatment were compared with baseline and compared to X-ray (4). (C) The Weighted Molecular Distance to Health calculated for the transcriptional signature is shown against the time after treatment (A–C From Berry *et al*., *Nature* 2010) (4). (D) A blood transcript signature is seen to change on treatment by as early as 1 week (12) ‘Cliff *et al*., Distinct phases of blood gene expression pattern through tuberculosis treatment reflect modulation of the humoral immune response. (From Cliff *et al*., *J Infect Dis* 2013; 207(1): pp. 18–29, by permission of Oxford University Press’ (E) A 664 transcript signature is seen to change on treatment by as early as 2 weeks (From Bloom *et al*., *Plos One* 2012) (11).

**Figure 4:**
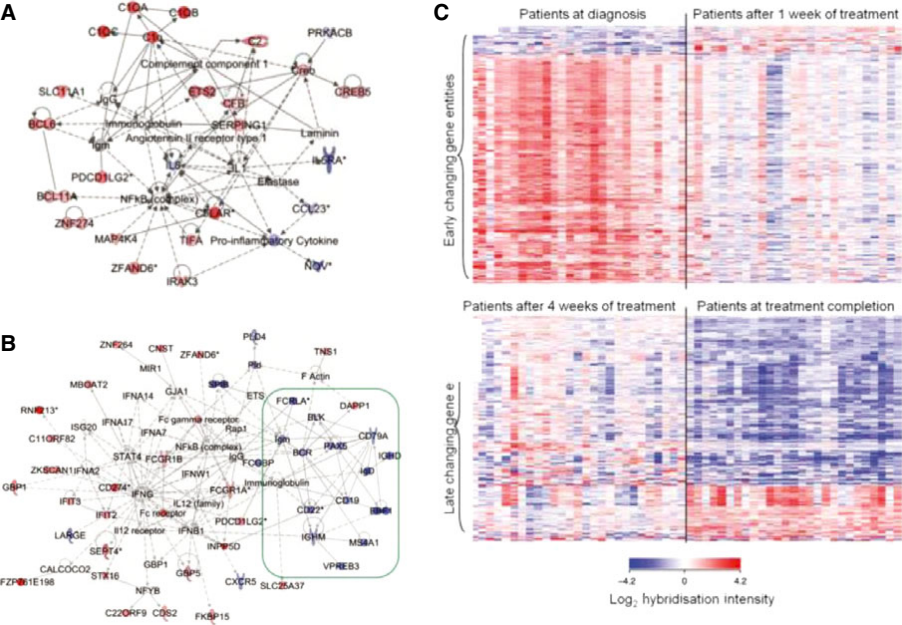
The blood transcriptional signature changes during different phases of treatment. Pathway analysis of the blood transcriptional signature reveals (A) early changes in complement related genes after 1 week; (B) later changes of other gene sets and restoration of T and B-cell related genes. (C) There is heterogeneity in the rate of change of gene expression between patients, but large-scale similarity in the overall pattern of change. (From Cliff *et al*., Distinct phases of blood gene expression pattern through tuberculosis treatment reflect modulation of the humoral immune response. *J Infect Dis* 2013; 207(1): pp. 18–29, by permission of Oxford University Press).

Monitoring tuberculosis drug efficacy is important for both clinical management and the development of new treatments. Berry *et al*. (4) observed that the blood transcriptional signature of active tuberculosis, consisting largely of increased IFN-induced gene expression, diminished by 2 months of treatment and had disappeared by 12 months after diagnosis (*Fig*.3*A*). This change in the molecular signature paralleled the clinical improvement measured by chest X-ray (*Fig*.3*B*) and was reflected in the MDTH score measured for individual patients (*Fig*.3*C*). The diminished T and B-cell signature of active tuberculosis also reverted in some individuals after 2 months of treatment, but this change was most apparent in all individuals by 12 months post-treatment (*Fig*.3*A*). In two separate subsequent longitudinal studies based in South Africa (11, 12) and London (11), it was apparent that large-scale changes in blood host gene expression occur very rapidly after initiation of successful tuberculosis treatment (*Fig*.3*D and E*), with differences detectable after only 1 week of treatment (*Fig*.4). Slower changes continued to occur throughout the duration of tuberculosis treatment (11, 12). Strikingly, similar results were obtained from both longitudinal studies, despite being conducted by different investigators using different technological platforms (*Figs*3*D, E and* 4). Pathway analysis showed certain genes changed expression immediately upon treatment initiation whereas others changed at later times, indicating that different biological pathways may predominantly be affected during different stages of treatment and recovery. A rapid downregulation of genes involved in the inflammatory response including a dominant C1q complement element occurred first at 1 week postinfection (*Fig*.4*A and C*) and was followed by changes in lymphocyte compartments with distinct upregulation of B-cell-related genes (12) (*Fig*.4*B and C*). Ottenhoff *et al*. (10) also observed temporal changes in blood gene expression through treatment, in samples collected from Indonesian tuberculosis patients (10), with expression patterns after 8 weeks being intermediate to the profiles obtained at tuberculosis diagnosis and at the end of treatment. Samples from healthy control subjects were similar to the samples collected at the end of tuberculosis treatment.

### Using changes in blood gene expression to monitor tuberculosis treatment

There is a great need for new tuberculosis drugs or regimens to shorten treatment and cope with drug resistance. The lack of easy and economical methods to test tuberculosis drug efficacy is a serious impediment to drug development. In later phase III drug efficacy trials, the rate of tuberculosis relapse over 2 years post-treatment is measured, and the proof of enhanced or non-inferior drug efficacy compared to existing drug regimens will require enrolment of many thousands of patients at vast expense. Currently, bacteriological approaches, such as the early bactericidal assay and the sputum conversion rate, are the most commonly used methods employed in early clinical studies. Although they provide some indication of early drug efficacy, particularly against actively replicating bacilli, they are not sufficient to predict tuberculosis treatment outcome and do not measure the killing of quiescent or persistent bacterial populations, which could later reactivate.

Host-derived biomarkers that predict tuberculosis treatment outcome could greatly enhance tuberculosis drug development (41). Radiological studies using PeT and CT in cynomolgus macaques can directly demonstrate drug efficacy at the level of individual granulomas, with different compounds shown to elicit different metabolic effects (42). The translation of such methodologies to human patients may allow early efficacy testing of tuberculosis regimens. However, a simple blood test would be much more easily applied in large-scale drug efficacy trials and may also be beneficial for clinical management. We therefore envisage that a test could be developed based on the blood gene expression changes that occur during successful tuberculosis treatment with conventional first-line four drug therapy consisting of isoniazid, rifampicin, pyrazinamide, and ethambutol. Furthermore, such a test could include genes whose expression changes both immediately upon treatment initiation, including complement and IFN-associated genes, to measure drug activity potentially against rapidly dividing bacilli, and also those transcripts that change expression more slowly, such those encoding B and T-cell genes, potentially reflecting the restoration of the normal gene expression as slower growing persistent mycobacterial populations are eradicated or leukocyte populations are restored. This approach would enable the test to measure the efficacy of compounds or regimens aimed at a wide range of mycobacterial targets.

To be useful across human populations, the test would need to include genes whose expression changes are consistent in different ethnic groups and in patients in different geographical locations. Furthermore, the test should also work in HIV^+^ as well as negative individuals. These issues are being addressed widely in further blood transcriptomic studies conducted in different geographical sites. It will also be important to characterize changes in blood gene expression that occur during non-standard treatments, such as second-line drug treatments used in multi-drug resistant tuberculosis.

The development of a test that reflects a successful and lasting cure would be beneficial for clinical management of tuberculosis patients. The response to tuberculosis treatment varies between individuals, which may be reflected by the blood signature (11, 12), and some risk factors for increased risk of tuberculosis-relapse after treatment completion exist. In this regard, people with type 2 diabetes are not only more likely to develop active tuberculosis upon infection than non-diabetic people (43) but also are more likely to suffer tuberculosis-relapse after treatment, treatment failure, or even death (44). Prediction of successful tuberculosis treatment in this population would be particularly valuable, and to this end patients are being recruited in different studies worldwide in patients with both tuberculosis and type 2 diabetes, which may help to unravel the causal relationship between the two diseases.

## Other methodologies with potential for diagnosis of tuberculosis

Although gene expression biomarkers from whole blood RNA have been widely applied to characterize differences between individuals with active tuberculosis and those with latent infection or other diseases, other complementary avenues may also be of use to support the analysis of unique parameters perturbed during tuberculosis disease. While it is understood that whole blood RNA expression reflects changes in peripheral blood cells migrating from infected tissue sites, additional and complementary information can be gained by measuring molecules that are derived directly from the site of infection (9, 45, 46). Studies systematically profiling the host response have employed numerous different methods. Proteomic studies have suggested that combinations of plasma or serum proteins can differentiate individuals with active tuberculosis from those latently infected with *M. tuberculosis* (47), from other diseases (48), and reflect changes during successful treatment (38, 49). There have been differing reports on whether cytokines will be of use in treatment monitoring (reviewed in 1), possibly due to instability of cytokines/chemokines due to post-transcriptional and post-translational regulation/degradation (50).

**Figure 5:**
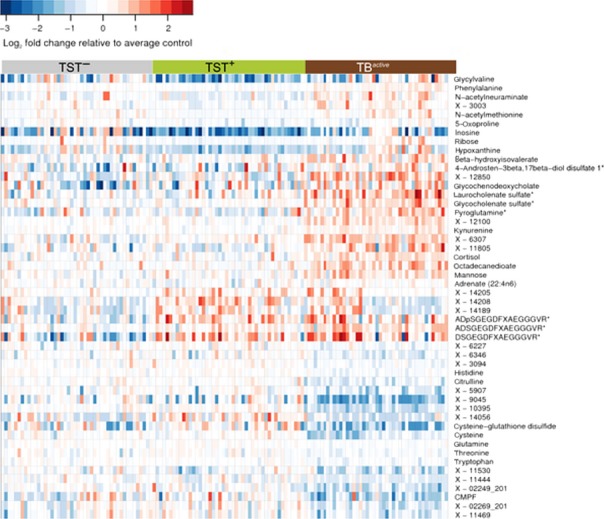
Metabolomics uncovers a signature of active tuberculosis in the blood reflecting immunosuppression. Heat map showing fold changes of small metabolic compounds in the three study groups, tuberculosis patients, healthy uninfected, and latently infected individuals. Fold changes are relative to the average abundance in the tuberculin skin test (TST) – group. Red indicates relative abundance higher than average in the TST– group; blue indicates relative abundance lower than average in the TST– group. Horizontal axis: samples belonging to the three study groups; vertical axis: top 50 compounds selected by variable importance in RF analysis, including compounds that could not be identified, but were strong predictors of sample status. Color bars above the heat map denote study groups: gray, TST–; green, TST+; red, TBactive. (From Weiner *et al*., *Plos One* 2012) (52).

Metabolomic profiling of serum has the potential to discriminate patients with active tuberculosis from latently infected individuals (51) and has highlighted a potential pathogenic role for tryptophan metabolism (52), which has previously been linked with mortality in tuberculosis (53) and may allow therapeutic limitation of inflammation (46). Specifically, Weiner *et al*. (52) analyzed metabolic profiles in serum samples of tuberculosis patients and healthy controls and showed that a biosignature based on profiles comprising less than 20 metabolites can achieve sensitivity and specificity (*Fig*.5), although this will need validation in further cohorts. Some of the predictive metabolites were suggested to have derived directly from the site of infection. For example, kynurenine, which was found at significantly higher levels in the sera of tuberculosis patients than in controls, is synthesized from tryptophan by the enzyme indoleamine 2,3-dioxygenase 1, induced in macrophages and dendritic cells in contact with *M. tuberculosis*, and it is possible that high kynurenine abundance in serum reflects release of this molecule from granulomas in the lung. Several processes comprising changes in their metabolic components (including higher levels of kynurenine and cortisol and lower abundance of lysophosphatidilcholines, which may be caused by inhibition of phospholipase A2) can be linked to immunosuppressive mechanisms. In a parallel approach, cytokines were determined in the same study groups (52).

Distinct cytokines correlated with defined metabolites, showing the link between metabolite abundance in serum and the immune processes in response to tuberculosis disease. For example, the chemokine C-X-C motif chemokine 10 (CXCL10, IP-10), the cytokine interleukin 6, and the growth factor granulocyte colony-stimulating factor showed a negative correlation with abundant amino acids, including glutamine and tryptophan, and positive correlation with metabolic markers elevated in the sera of tuberculosis patients, including N–acetylneuraminate and hypoxanthine. It will be important that these findings are validated in independent cohorts (51, 52) and in different locations, as has been done in the case of blood transcriptomics, to avoid overestimating diagnostic accuracy, especially with small sample sizes (54).

A microRNA-223 (miR-223) has been identified as an upregulated small non-coding RNA in blood and lung parenchyma of tuberculosis patients and during experimental mouse models of the disease (55). Deletion of miR-223 rendered mice highly susceptible to acute lung infection and it was suggested that miR-223 directly targets the chemoattractants CXCL2, CCL3, and IL-6 in myeloid cells. This study revealed an essential role for a single miRNA in tuberculosis and also identified new targets for and assigned biological functions to miR-223. Although in an early stage, the potential of miRs for diagnosis and therapy of tuberculosis is being considered (56). Likewise, there is discussion of the potential use of epigenetic markers as diagnostic markers in adverse immune system conditions and treatment thereof (57), although again much is to be uncovered as to the specificity and effectiveness of such approaches. Recent studies on epigenetic programming in differentiation of mononuclear phagocytes point to a role of epigenetics in innate immunity, which has been claimed to play a role in the non-specific prevention of morbidity and mortality by the tuberculosis vaccine BCG (58–60).

## What did we learn about the immune response from the transcriptomic analysis of active TB?

Although TNF, IL-12, and the Th1 axis have shown to be important in both mouse and human for protection against tuberculosis and other mycobacterial diseases (reviewed in 1), it remains unclear what constitutes a protective immune response (61) and what factors contribute to the development and progress of active tuberculosis disease.

**Figure 6:**
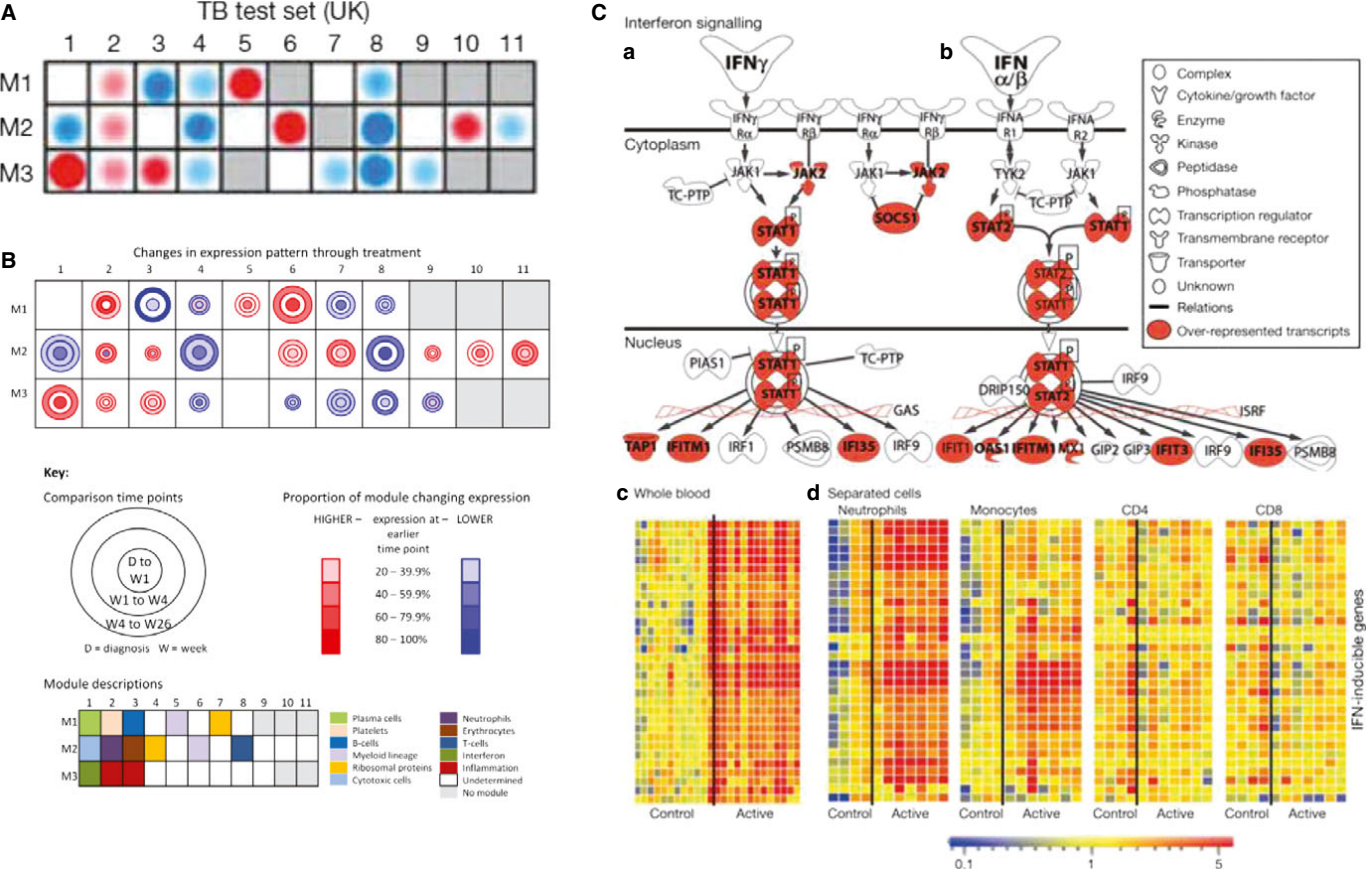
Modular and pathway analysis reveal a dominant IFN-inducible signature in tuberculosis. Modular analysis reveals overabundance of IFN-inducible genes (M3.1) and myeloid genes (M1.5 and M2.6) and under abundance of B (M1.3) and T cell (M2.8) related genes (A) (4); (B) (From Cliff *et al*., Distinct phases of blood gene expression pattern through tuberculosis treatment reflect modulation of the humoral immune response. *J Infect Dis* 2013; 207(1): pp. 18–29, by permission of Oxford University Press). (12). (C) The canonical pathway for IFN signaling from Ingenuity Pathways Analysis; with transcripts over-represented in test set patients with active tuberculosis (1, 4) shaded red. GAS, Gamma-activated site; ISRE, IFN-sensitive element. Modified from (1, 4). Transcript abundance in whole blood and (c) separated blood leucocyte populations of representative IFN-inducible genes (from top to bottom: OAS1, IFI6, IFI44, IFI44L, OAS3, IRF7,IFIH1, IFI16, IFIT3, IFIT2, OAS2, IFITM3, IFITM1, GBP1, GBP5, STAT1, GBP2, TAP1, STAT1, STAT2, IFI35, TAP2, CD274, SOCS1, CXCL10, IFIT5) in active tuberculosis. Transcript abundance/expression is normalized to the median of the healthy controls. Modified from (A and C modified from Berry *et al*., *Nature* 2010) (1, 3, 4).

Transcriptomic analyses of blood from patients with tuberculosis has revealed a dominant signature of IFN-inducible genes, including those down-stream of type I and type II (IFN-γ) signaling (4, 8, 10, 12), upregulation of complement related genes (12) and those associated with myeloid function and inflammation (4, 7, 8, 11, 12, 21, 62) (*Fig*.6). In addition, the signature reflects downregulation of genes encoding B and T-cell functions (4, 12, 21) (*Fig*.6), the latter accounted for by decreased numbers of T cells in the blood of active tuberculosis patients (4). This perhaps reflects apoptosis of such cells in the blood or their migration to the infected tissue (4).

### Potential mechanisms whereby type I IFN contributes to pathogenesis in tuberculosis and other mycobacterial diseases

The blood transcriptomic approach identified a previously unappreciated signature of tuberculosis dominated by type I IFN, suggesting a potential role of Type I IFN in contributing to active tuberculosis disease (4, 8, 10, 12). This was supported by the observations that the signature correlated with the radiographic extent of disease (4) and was diminished upon treatment (4, 11, 12). Through microarray analysis of human tissue from patients with the mycobacterial disease leprosy (24) and comparison with deposited data on the blood transcriptional signature of active tuberculosis (8, 23), IFN-inducible transcripts have also been reported in leprosy. Using these expression data and supported by *in vitro* models, this study suggested that in leprosy the balance of type I and type II IFNs determines a protective or susceptible host immune responses (24). Likewise, in experimental models of tuberculosis, type I IFN has been shown to have adverse effects (63–66), although there are conflicting reports (67). It is of note that adverse effects of Type I IFN were invariably reported in experimental models of tuberculosis where mice were infected with clinical isolates of *M. tuberculosis* as opposed to the laboratory strain H37Rv that is largely used. In addition, the experimental mouse models were on a genetic background other than the C57Bl/6 or BALB/c that are widely used as experimental models of tuberculosis (63–65, 68). Elevation of type I IFN by *M. tuberculosis* infection of mice in the context of Poly(I)C (69, 70) or during coinfection with influenza (71) or through genetic manipulation removing negative regulation of type I IFN expression by the MAP-kinase TPL-2 (72) results in exacerbated disease and increased bacterial load, all via a type I IFN-dependent mechanism.

The mechanisms that mediate IFNα/β-driven disease exacerbation are not fully understood and will undoubtedly be complex. Data from investigations of hyper-virulent strains initially suggested suppression of pro-inflammatory cytokines and Th1-type immunity (63, 65). Recent data suggest that type I IFN enhanced migration of inflammatory monocytes and neutrophils to the lung in *M. tuberculosis-*infected mice (68). In keeping with effects on myeloid cells, recent mechanistic studies have shown that type I IFN can induce the suppressive cytokine IL-10 (24, 70, 73) and inhibit the production of the cytokines IL-12 and TNF important for protection against *M. tuberculosis* infection (24, 70, 73). Cell-intrinsic type I IFN signals additionally suppressed iNOS production by pulmonary myeloid cells (70). The induction of immune suppressive IL-10 (reviewed in 1, 74) and IL-1RA by IFNα/β may be part of the mechanism for this suppression of protective pro-inflammatory cytokines (24, 70, 72, 73). Additionally, type I IFN can block the ability of IFN-γ (type II IFN) to activate macrophages to control bacterial growth or produce the protective cytokine IL-12 (24, 73), and hence, the balance of induction of these two cytokines is critical for the outcome in infection (24, 73). Finally, polarization toward myeloid-derived suppressor cells, which counteract protective immune responses against *M. tuberculosis*, needs to be considered (75). Ablation of myeloid-derived suppressor cell effects by all-trans-retinoic acid in a susceptible mouse model of tuberculosis points to the potential of host-directed therapy of aberrant myeloid-derived suppressor cell responses as an adjunct to conventional drug treatment of tuberculosis (75).

IL-1α and IL-1β have been shown recently to inhibit type I IFN induction in mouse and human macrophages, and when IL-1 was present in type I IFN-treated cultures, it also suppressed the pro-bacterial effects downstream of IFNβ. IL-1-induced prostaglandin E2 (PGE2) was also able to potently inhibit type I IFNs in this context (76). Moreover, targeting PGE2 during *M. tuberculosis* infection, either via direct administration of the prostanoid or enhancement by 5-lipoxygenase blockade with zileuton reversed polyI:C-mediated type I IFN-driven mortality (76). This has major implications for the use of zileuton for host-directed therapy to improve and possibly speed-up the current drug treatment of tuberculosis (76). The use of the blood transcriptome (4, 11, 12) to monitor effective treatment is likely to be a good supportive approach in clinical trials that utilize these immunomodulators.

Although high levels of type I IFN may be detrimental to the outcome of *M. tuberculosis* infection, it is likely that low levels at the initiation of an immune response, or in particular settings, may be protective against this pathogen. For example, in experiments using *Ifngr*^−/−^*Ifnar1*^−/−^ mice, IFNα/β is suggested to contribute to host protection in the absence of the IFNγ pathway (77). Furthermore, naturally occurring mutations in host-protective *ISG15* in humans suggests that IFNα/β can induce host-protective responses to mycobacterial infection, although it is not clear under what circumstances IFNα/β induces this gene during *M. tuberculosis* infection (78). Regardless, many studies demonstrate a dominant IFN-inducible signature of active tuberculosis (24, 63–65, 70–73, 76), and it is possible that Type I IFN may contribute to disease progression, since it is higher in patients with the greatest radiographic lung disease (4) and is diminished upon effective treatment (4, 11, 12).

## Concluding remarks

**Figure 7:**
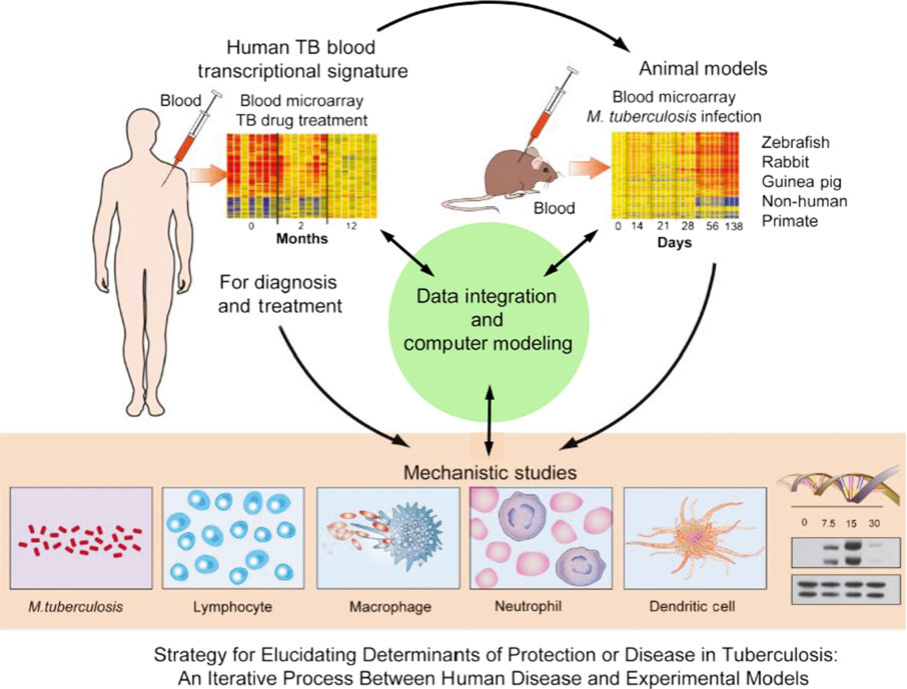
Using a systems biology approach in infectious diseases. This figure defines the strategy for elucidating determinants of protection or disease in tuberculosis: an iterative process between human disease and experimental models. Modified from O'Garra *et al*., 2013 (1); O'Garra *et al*., 2013 (80); Berry *et al*., 2013 (3); Blankley *et al*., 2014 (5).

Evidence to date demonstrates that blood transcriptomics present a robust approach for studying the immune response in tuberculosis and other diseases, as similar findings have now been reported from various groups in different geographical locations. The findings offer information as to the immune response underlying the pathogenesis of tuberculosis and may provide tools toward diagnosis, treatment monitoring, and in the development of host-directed therapy regimens to support drug treatment. The potential use of blood transcriptomics in the clinical management of tuberculosis is likely to support current diagnostic tests and may help to speed up diagnosis and treatment, thus reducing transmission. The development of clinic-friendly tools for supporting the diagnosis of tuberculosis and treatment monitoring have been suggested to rely on a set of discriminant classifiers (consisting of between 12 and 100 genes) that would be easily convertible into a PCR-based affordable assay, allowing its development in the clinic for tuberculosis diagnosis. Meta-analyses of diverse studies on the blood transcriptome of tuberculosis, in the context of other infectious or non-communicable diseases, are required to determine the unifying signature reflecting tuberculosis versus other diseases and the blood signature for tuberculosis treatment success. It is possible with advancing technology and decreasing pricing that a composite whole genome expression profile, obtained using either microarray or in the future RNA-Seq, may be useable rather than discriminant classifiers. This will require the development of sophisticated tools for rapid integration of complex host transcriptional signatures, the clinical data and the pathogen identification, and storage and easy access to such data. Advancement of bioinformatics tools (37) will also help in uncovering biological pathways underlying disease susceptibility, including co-infection, and also help to uncover the comorbidities caused by infectious and non-communicable diseases. This will require iterative research approaches between experimental models and human disease (1, 3, 5, 79) (*Fig*.7) to establish the function of immune and inflammatory pathways and molecules in resistance or susceptibility to *M. tuberculosis* infection.
